# PD20 Economic Assessment And Budget Impact Model Of Injectable Ganciclovir For Cytomegalovirus Infection In Immunosuppressed Patients In Brazil

**DOI:** 10.1017/S026646232400285X

**Published:** 2025-01-07

**Authors:** Bárbara Rodrigues Alvernaz dos Santos, Álex Martins, Ana Clara Silva Mendes, Camila Pereira, Livia Nassif, Ludmila Gargano, Isabela Freitas, Roberto Muniz-Júnior, Francisco de Assis Acurcio, Juliana Alvares-Teodoro, Augusto Guerra-Junior

## Abstract

**Introduction:**

Cytomegalovirus (CMV), a prevalent human herpes virus, has long periods of latency. It can reactivate following immunosuppression causing diverse clinical manifestations. In immunocompetent individuals, CMV infections are usually asymptomatic. However, in immunosuppressed individuals, such as patients with human immunodeficiency virus, CMV infections can cause serious complications. Antiviral treatment is crucial for minimizing health risks and preventing severe outcomes, such as retinitis and colitis, in this population.

**Methods:**

A cost-effectiveness analysis was conducted using a decision tree model to estimate the incremental cost-effectiveness ratio (ICER) of ganciclovir (1 mg/mL) for people with acquired immunodeficiency syndrome (AIDS) affected by CMV-related retinitis or colitis. The model compared ganciclovir therapy to no ganciclovir, with the outcome being progression of CMV disease. Budget impact was considered over a five-year time frame with 100 percent diffusion among patients with AIDS and CMV-related colitis.

**Results:**

In patients with AIDS and CMV-related retinitis, use of ganciclovir incurred an incremental cost of BRL29,378.95 (USD5,188.70) with an incremental effectiveness of 0.51 in the therapeutic response rate and an ICER of BRL57,605.79 (USD10,173.93). For those with AIDS and CMV-related colitis, ganciclovir incurred an incremental cost of BRL10,040.71 (USD1,773.32), with an incremental effectiveness of 0.26 and an ICER of BRL38,618.11 (USD6,820.45). The estimated cumulative budget impact, considering 100 percent ganciclovir diffusion over five years, was BRL1,124,800,751.17 (USD198,654,342.23) and BRL571,223,309.75 (USD100,885,415.26) for patients with AIDS CMV-related retinitis and colitis, respectively.

**Conclusions:**

The application of economic evaluation and budget impact tools is crucial for maintaining health system sustainability. Introducing treatments with high ICERs and budget impact can jeopardize the health system and access to treatments. Upholding criteria, such as the willingness-to-pay threshold, is essential for ensuring the sustainability and continuity of health care.

